# *MatureBayes*: A Probabilistic Algorithm for Identifying the Mature miRNA within Novel Precursors

**DOI:** 10.1371/journal.pone.0011843

**Published:** 2010-08-06

**Authors:** Katerina Gkirtzou, Ioannis Tsamardinos, Panagiotis Tsakalides, Panayiota Poirazi

**Affiliations:** 1 Computer Science Department, University of Crete, Heraklion, Greece; 2 Institute of Computer Science (ICS), Foundation for Research and Technology-Hellas (FORTH), Heraklion, Greece; 3 Institute of Molecular Biology and Biotechnology (IMBB), Foundation for Research and Technology-Hellas (FORTH), Heraklion, Greece; Baylor College of Medicine, United States of America

## Abstract

**Background:**

MicroRNAs (miRNAs) are small, single stranded RNAs with a key role in post-transcriptional regulation of thousands of genes across numerous species. While several computational methods are currently available for identifying miRNA genes, accurate prediction of the mature miRNA remains a challenge. Existing approaches fall short in predicting the location of mature miRNAs but also in finding the functional strand(s) of miRNA precursors.

**Methodology/Principal Findings:**

Here, we present a computational tool that incorporates a Naive Bayes classifier to identify mature miRNA candidates based on sequence and secondary structure information of their miRNA precursors. We take into account both positive (true mature miRNAs) and negative (same-size non-mature miRNA sequences) examples to optimize sensitivity as well as specificity. Our method can accurately predict the start position of experimentally verified mature miRNAs for both human and mouse, achieving a significantly larger (often double) performance accuracy compared with two existing methods. Moreover, the method exhibits a very high generalization performance on miRNAs from two other organisms. More importantly, our method provides direct evidence about the features of miRNA precursors which may determine the location of the mature miRNA. We find that the triplet of positions 7, 8 and 9 from the mature miRNA end towards the closest hairpin have the largest discriminatory power, are relatively conserved in terms of sequence composition (mostly contain a Uracil) and are located within or in very close proximity to the hairpin loop, suggesting the existence of a possible recognition site for Dicer and associated proteins.

**Conclusions:**

This work describes a novel algorithm for identifying the start position of mature miRNA(s) produced by miRNA precursors. Our tool has significantly better (often double) performance than two existing approaches and provides new insights about the potential use of specific sequence/structural information as recognition signals for Dicer processing. Web Tool available at: http://mirna.imbb.forth.gr/MatureBayes.html

## Introduction

MicroRNAs (miRNAs) are small, usually 19–27 nucleotides long, single-stranded RNAs that are generated from endogenous hairpin shaped transcripts [1]. MicroRNAs function as regulatory molecules in post-transcriptional gene silencing by base pairing with target mRNAs, leading to mRNA cleavage or translational repression, depending on the degree of complementarity between the miRNA and its target transcript.

Although miRNAs are functionally similar to short interfering RNAs (siRNAs), they are unique in terms of their biogenesis. MicroRNA genes are most likely transcribed by RNA polymerase II into pri-miRNAs which are long, double-stranded, unstructured precursors with a cap on the 5′ end and a Poly(A) tail on the 3′ end [2], [3]. In most cases, the pri-miRNA is enzymatically processed by the Microprocessor complex (Drosha and cofactor DGCR8/Pasha) into the precursor miRNA (or pre-miRNA), a stem-loop structure of about 60–100 nucleotides with a 2 nucleotide overhang on the 3′ end [4].

In mammals, pre-miRNAs are transported to the cytoplasm by Exportin-5, a nucleus export factor, in a Ran-GTP dependent manner [5], [6]. After being exported from the nucleus, pre-miRNAs are processed into approximately 22 nucleotide long miRNA duplexes with a 3′ 2 nucleotide overhang by the cytoplasmic RNase III, Dicer [7]. Dicer is a highly conserved protein that is found in almost all eukaryotic organisms. Following the pre-miRNA processing by Dicer into a miRNA:miRNA* duplex, one (or both) of the RNA strands is incorporated into RISC for target recognition. RISC is composed of Dicer, Argonaute (AGO) and other non-specified proteins. The functional (or mature) miRNAs base-pair with their mRNA targets, leading either to mRNA degradation, if there is sufficient complementarity between the miRNA and the target mRNA, or to translational repression [8], [9].

A large body of experimental findings indicates that the regulatory action of miRNAs is essential for most organisms as these tiny molecules play a central role in processes like developmental timing [10], apoptosis [11], cell proliferation and differentiation [12], [13], as well as numerous diseases (for a review see [14]) and anti–viral defense [15]. Thus, over the last decade, significant amount of effort has been devoted to finding and characterizing the function of miRNAs across multiple organisms [16]–[20].

The main experimental approaches for the identification of mature miRNAs include forward genetics (traditional cloning) and the use of small RNA libraries [16]–[20], both of which suffer from numerous shortcomings. A common limitation of all cloning approaches is the difficulty to find miRNAs that are expressed at low levels and/or specific tissues or developmental stages. Moreover, certain miRNAs may be hard to clone due to physical properties such as sequence composition, or to post-transcriptional modifications, such as editing or methylation [16]. Forward genetic approaches on the other hand are relatively inefficient due to the small size of miRNAs and their potential tolerance to mutations that do not affect the “seed” region. Such mutations make miRNA genes difficult-to-hit targets in spontaneous or induced mutagenesis. Since the seed region (positions 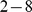 of the miRNA) is critical for finding respective gene targets, accurate identification of the start position of the mature miRNA within a miRNA precursor is of major importance.

A number of computational methods have recently been developed to counteract these limitations and complement experimental approaches (for a review see [20]). Most of these methods, however, focus on the discovery of either novel miRNA genes in the genomes of various species or possible mRNA targets of the known miRNAs [19], [21]. On the contrary, few attempts have been made to computationally predict the functional part of the miRNA precursor, namely the mature miRNA [22]–[26]. More importantly, existing tools suffer from a number of shortcomings which limit their applicability. These include inaccurate hypotheses, such as the assumption that every hairpin structure produces just a single mature miRNA [22], [23] or that pri–miRNAs are always processed by the Drosha complex, whose cleavage cite determines the start position of the mature miRNA [24], [27]. Evaluation of performance is also problematic as it is often measured in terms of true positive rate alone, ignoring the number of false positives [25], [26].

In this work we introduce a computational method, called *MatureBayes*, that uses a Naive Bayes Classifier (NBC) to predict the start position of the mature miRNA on human and mouse miRNA precursors. The generalization ability of the model on experimentally verified miRNAs from two other species (*Drosophila melanogaster* and Zebrafish) is also assessed. It should be noted that precursors downloaded from miRBase do not necessarily correspond to the actual miRNA precursors. Specifically, each entry in the miRBase Sequence database represents a predicted hairpin portion of a miRNA transcript, with information on the location and sequence of the mature miRNA sequence. In this work we use only experimentally verified mature miRNAs and their corresponding precursors. The model utilizes information about the sequence and structure of miRNA precursors and takes into account both positive and negative examples in order to identify the start position of either the mature miRNA(s) (assuming the functional strand is known) and/or the miRNA:miRNA* duplex. The importance of specific positions along the miRNA precursor sequence as predictive features and their potential role in Dicer processing is also investigated. Comparison with existing tools is performed on a common blind set by contrasting the respective distance distributions of the computational predictions from true mature miRNAs.

## Materials and Methods

### Datasets

Experimentally verified human and mouse mature miRNAs from the miRBase database (version 14) (http://www.mirbase.org) were used to train and evaluate our model. Human and mouse data were combined in order to generate a large enough dataset for optimizing the model's performance. The training set consisted of 533 human precursors producing 729 mature miRNAs and 422 mouse precursors producing 530 mature miRNAs, respectively (miRBase database version 10.1). The evaluation dataset (hereby termed Test Set I) consisted of 188 human precursors producing 197 mature miRNAs and 141 mouse precursors producing 148 mature miRNAs, respectively. There was no overlap between the evaluation and training sets as the latter contained miRNAs added in versions 11–14 of miRBase database. Moreover, precursor sequences in the evaluation set had low similarity (on average 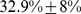) with the sequences used in the training set, in an attempt to avoid over-fitting. To test our model's generalization performance on other species, a second evaluation data set (hereby termed Test set II) was also used, consisting of 218 Zebrafish precursors producing 253 mature miRNAs and 51 *Drosophila melanogaster* precursors producing 54 mature miRNAs, respectively. This dataset consisted of miRNAs (mirBase database version 14) whose mature sequences have been experimentally verified in the species of interest (Zebrafish or *Drosophila melanogaster*) and at least one other organism listed in miRBase, using the search algorithm *blastn* with evalue 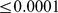 as a similarity criterion.

Overall, only experimentally verified mature miRNAs were used to form the positive class in both training and evaluation datasets. Negative examples were generated from the respective precursor sequences based on the observation that known miRNA precursors do not produce multiple overlapping mature miRNAs from the same arm of the fold-back precursor [28]. Specifically, for each verified mature miRNA, we used a same-size sliding window and selected all possible sequences which could be created by sliding 1 base pair towards either direction from the verified mature miRNA over the precursor sequence, excluding any hairpin loops. This procedure resulted in a very large negative set, where each mature miRNA had a variable number of corresponding negatives, depending on the number of precursors that produce this miRNA and their length. To reduce execution time while maintaining a good representation of the negative class, we decided to use a randomly selected subset of negative examples for each mature miRNA. Specifically, we used a ratio of 1 positive to 10 negative examples, as this was the largest ratio for which there was no change in the estimated probability distributions for the negative class features (see section Representation of Biological Features used in the Classifier).

### Naive Bayes Classifier

Naive Bayes is a simple probabilistic classifier which is based on the application of the Bayesian theorem with strong (naive) independence assumptions. Classification is performed by assigning each sample to the *a posteriori* most probable class, considering that the input features of a sample of any given class are conditionally independent given the class [29]. Specifically, the output of NBC is the ratio between the posterior probabilities of a sequence for belonging to the positive class versus the negative class. In this work, we primarily exploit the ranking capabilities of naive Bayes classifiers [30] rather than the classification ones, in order to provide the most probable mature miRNA candidate(s) within a miRNA precursor sequence. This is achieved by ranking all sliding window sequences within a precursor according to the NBC output and selecting the top ranking candidate (i.e. the Top Scorer) as the predicted mature miRNA (i.e. the computational truth).

The classification performance of the naive Bayes model is optimized according to the Area Under the Receiver Operating Characteristic (ROC) curve (AUC), using the threshold averaging algorithm introduced by Fawcett [31] during the cross-validation procedure. We use AUC as a measure of classification performance as it is insensitive to both skewed class distributions and unequal classification error costs [31] while it is not limited by a specific threshold for the classification of the data, thus enabling a better exploration of the ranking capabilities of the naive Bayes classifier [30]. AUC is used primarily for optimizing the various model parameters, while the prediction performance of the algorithm with respect to correct identification of the mature miRNA start position is evaluated using distance distributions between the predicted and actual mature miRNAs on all miRNA precursors. Distance distributions are generated by measuring the difference between the predicted and the actual start position of each mature miRNA in the test sets.

### Model Outputs

*MatureBayes* offers two alternatives for computing the most probable start position of the mature miRNA(s) in any given miRNA precursor. If the stem that produces a mature miRNA (or functional stem) is known, then the proposed computational truth is the top scoring candidate produced by the classifier for that specific stem. The complementary stem is not considered in this case. Alternatively, if the functional stem is not known, the proposed computational truth is the duplex formed by the top scoring candidate estimated over *both* stems, along with its miRNA*. A miRNA* is defined as the same-size mature miRNA candidate that lies on the opposite strand and starts 2 nucleotides away from the matching position of the mature miRNA candidate ending position, towards the 3′ end of the precursor, according to existing biological evidence [20]. Although there is evidence that miRNA* does not always conform to this definition, it is currently the most widely accepted definition that corresponds to the majority of miRNA duplexes. Note that the top scoring candidate of the entire precursor does not necessarily correspond to the top scorer of the functional stem or its miRNA*. It could be a completely different molecule. Thus, the two types of model outputs can generate different predictions.

The classifier's prediction accuracy for the two types of model outputs, i.e. the predicted mature miRNA and/or the predicted miRNA:miRNA* duplex is evaluated by generating distance distributions of the predicted start position from that of the closest actual mature miRNA on each precursor sequence. For the mature miRNA prediction, the distance distribution is estimated over the known functional stems, i.e. the stems known to produce a mature miRNA. For the miRNA:miRNA* duplex prediction, distances are calculated between the actual mature miRNA and the predicted mature miRNA or its miRNA*, depending on which one is located on the functional stem. If a precursor produces two mature miRNAs, both distances are calculated.

### Representation of Biological Features used in the Classifier

**Figure 1:**
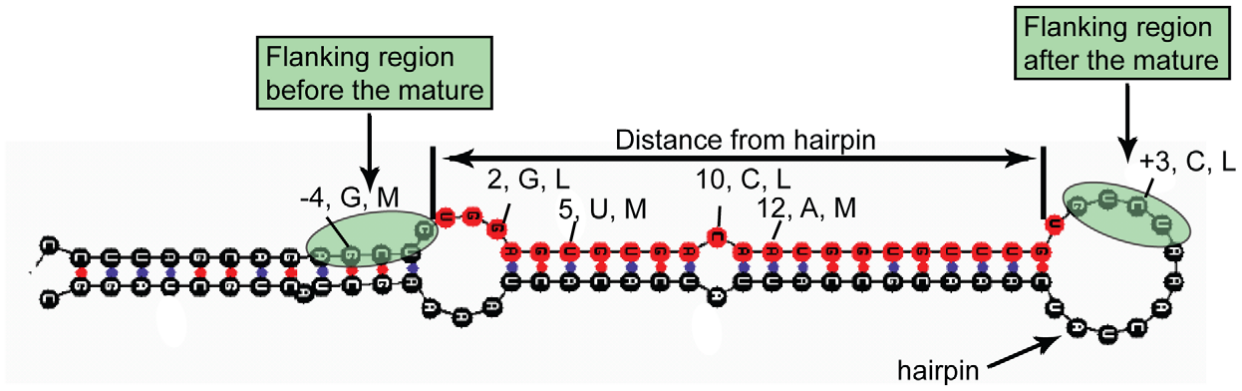
Illustration of the features used to describe positive and negative miRNA samples. The figure shows a 5′ mature miRNA sample (in red) and the associated flanking regions (in green). Examples of sequence and structural information for certain positions in the mature miRNA as well as the flanking regions are also depicted. The distance feature, measuring the number of nucleotides from the start position of the mature miRNA until the start of the closest hairpin is indicated on top.

The proposed model considers two types of biological features, namely sequence and structure of miRNA precursors, as illustrated in Figure 1. Specifically, each mature miRNA is represented as a 2-dimensional character array containing information about the base composition (Adenine, Cytosine, Uracil and Guanine represented as A, C, U and G, respectively) and structure (match or mismatch represented as M and L, respectively) for each position along the mature miRNA sequence. The same position-specific information is also considered for a flanking region of 9 nucleotides that extends symmetrically along both sides of the mature miRNA in the precursor sequence, where the size of the flanking region is selected to optimize classification performance on the training set (see Supplementary Table S1). The same representation is used to describe negative samples (which are generated by sliding along the precursor). In other words, each position along the input sequence (positive or negative) is represented by one of the following 9 pairs, corresponding to the 8 possible combinations of sequence and structure and the “noValue” pair
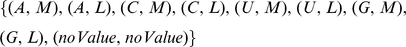
The “noValue” pair is used to indicate the lack of information on positions within the flanking region that may be located outside the limits of the precursor. For example, if positions ‘0–4’ of a given mature miRNA contain 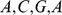 and 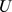, respectively and their structural information is 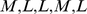, they would be represented as
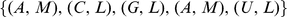
These features are termed position-specific features as they provide information about the sequence and structural characteristics of a given position along the mature miRNA within a miRNA precursor. The contribution of sequence versus structural information to the model's performance was investigated earlier, indicating that a combination of both is most informative for the specific problem [32]. In addition to the above position-specific features, the distance of the start position of each mature miRNA (and its respective negatives) from the closest hairpin of the precursor is also used as a characteristic input feature to the classifier.

### Parameter Optimization

There is a total of three free parameters in the model: (1) the size of the flanking region surrounding the mature miRNA 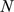, (2) the size of the scanning window 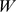 which is used to identify the mature miRNA candidates and (3) the number of position-specific features 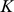 used to represent the positive and negative examples. The values for these parameters were optimized using 10-fold cross validation [33] over the training set and recording the AUC of each trained classifier. Specifically, all precursors in the training set were partitioned into 10 equal subsets, 9 of which were used for training the classifier, while the left out subset was used for validation. Performance on the validation set was estimated by producing and classifying negative and positive examples as in the training set, from the left-out miRNA precursors. This process was repeated iteratively until all data were used for both training and validation. It is important to note that the AUC is estimated based on exact match between the start position of the predicted versus the actual mature miRNA(s). Even 1nt deviations are considered as negative examples.

Six flanking region sizes (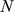 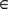 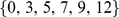) and four scanning window lengths (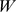 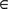 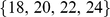) along with all possible position-specific features, were investigated. Note that 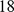 was the size of the smallest mature miRNA in our training set, and 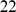 was the average size. Supplementary Table S1 shows that classification performance was maximized for a window of 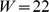 nucleotides and a flanking region of 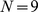 nucleotides while Supplementary Table S2 shows that a number of 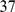 position features resulted in maximum classification performance.

### Feature Selection

In order to identify the positions within the input sequence which contain significant discriminatory information between positive and negative examples, we generate mass probability functions for each position-specific feature over the positive and negative classes and use the symmetric Kullback-Leibler divergence metric [34] to measure the difference between the respective distributions.

The Kullback-Leibler divergence (K-L divergence) is a measure of the difference between two probability distributions [35]. For Probability Mass Functions (PMFs) P and Q of a discrete random variable, the K-L divergence of Q from P is defined as:
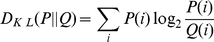
(1)


Note that the K-L divergence is not a true metric since it is not symmetric, namely 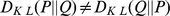. To overcome this problem we use the symmetric and non-negative Kullback-Leibler divergence [34], which is defined as:
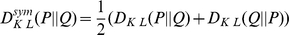
(2)and is commonly used in classification problems.

Feature selection in *MatureBayes* is performed according to the following procedure.

1. For each position-specific feature we generate the probability mass functions for both positive and negative examples in the training set.
2. Using the symmetric K-L divergence metric, we measure the difference between the probability mass functions for all position-specific features.
3. We rank the position-specific features according to the K-L score whereby large distances are considered more informative.
4. We then train the classifier using the top *K* features. Each feature is incorporated sequentially only if it improves the performance of the classifier measured as the Area Under the ROC curve.

**Figure 2:**
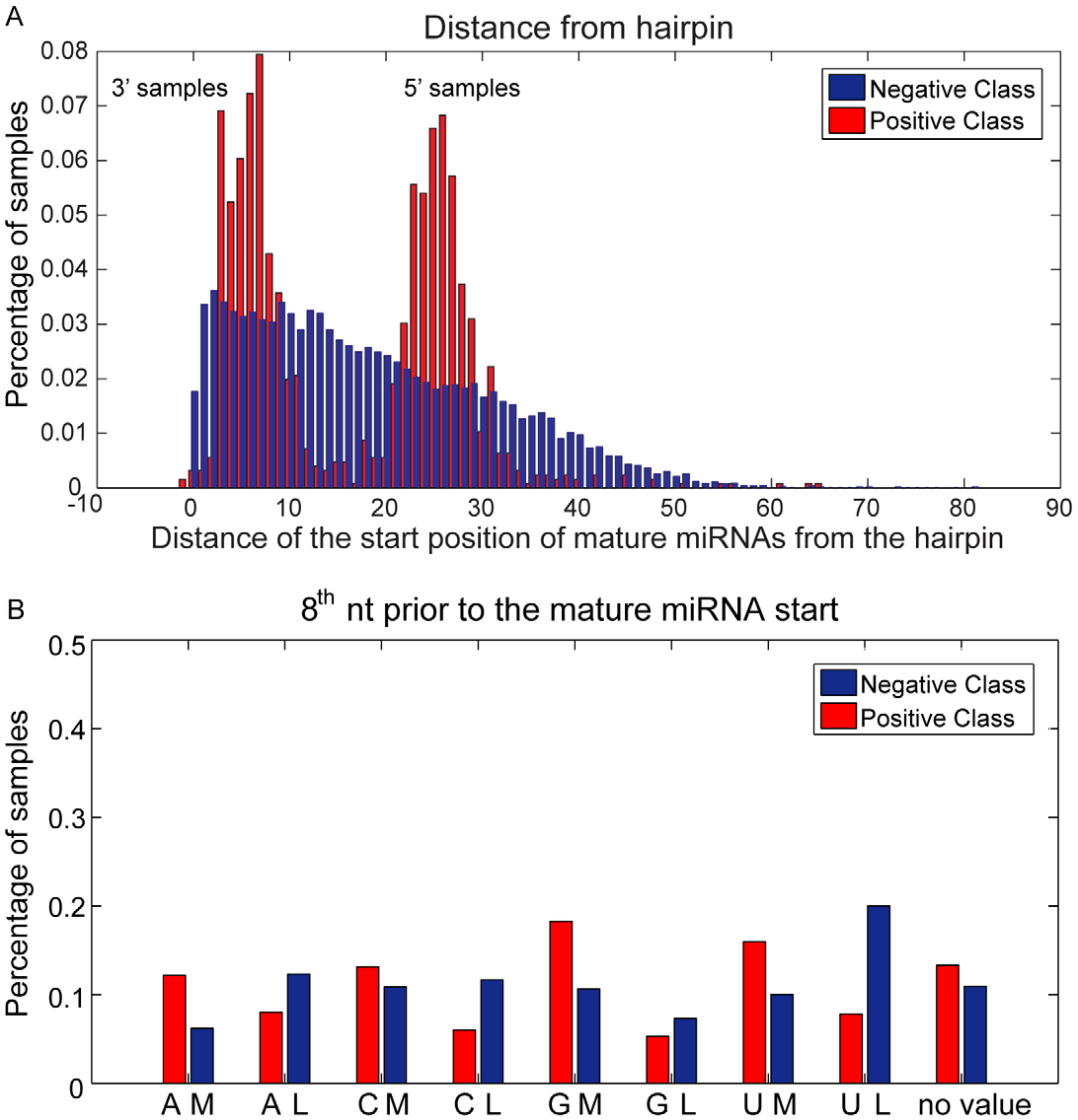
Class conditional probability distributions of the two top ranking input features. A. Combined distance-from-the-hairpin distribution for 3′ and 5′ miRNAs. Note that for 5′ miRNAs the distribution is shifted by approximately 22 nucleotides (average length of the mature miRNA) as the mature miRNA is located between the hairpin and the miRNA start position. This is not the case for the 3′ samples. Note that in both cases, the distribution of actual mature miRNAs is quite narrow indicating that mature miRNAs are located within a short distance from the hairpin. B. Distribution of the position-specific feature located 8 nucleotides prior to the start of the mature miRNA sample. Note that differences between positive and negative data are small, even for the top scoring position-specific feature, indicating that the two classes are hard to distinguish. All distributions are estimated over the training set.

Representative examples of the class conditional probability distributions taken over the training set for the two most important features are shown in Figure 2. Figure 2A shows the respective distributions for the distance between the start position of a mature miRNA sample and the closest hairpin. Distances were estimated separately for 3′ and 5′ samples and results were pooled together to form the combined distribution. As evident from the figure, this distance ranges within a small set of values in the positive class while for the negative class it can be described by a uniform distribution. The former suggests that true mature miRNAs are located within a close distance from the nearest hairpin, as previously suggested [5]. Note that the uniform distribution of negative data results from their generation process (see ‘Datasets’). Figure 2B shows an example of the respective class distributions for the top-scoring position-specific feature located 8 nucleotides prior to the start of the mature miRNA (position 8 in the 5′ flanking region). The specific feature ranked first according to the Kullback-Leibler metric during the feature selection process. As evident from the figure, the positive and negative class distributions are very similar, even for the top-scoring position-specific feature, making discrimination a very challenging task.

## Results

### Classification Performance

The model's classification performance is optimized using a 10-fold cross validation procedure in which the classifier is iteratively trained on positive and negative mature samples and evaluated against the precursors corresponding to the left-out mature miRNAs. Generalization performance is then assessed using two blind test sets. Specifically, a total of 955 human and mouse precursors generating 1259 mature miRNAs are used for training (cross-validation), while 329 human and mouse precursors corresponding to 345 mature miRNAs are used for testing (Test set I). An additional set of 269 Zebrafish and *Drosophila melanogaster* precursors generating 307 mature miRNAs with multiple experimental support (see ‘Datasets’) are used to test our method's generalization performance with respect to other species (Test set II). Performance is estimated using an optimized sliding window of 22 nucleotides (see ‘Parameter Optimization’ in the Materials and Methods section), whereby all possible mature miRNA candidates, generated by sliding the window one base pair in both stems of each queried precursor apart from the hairpin loop(s), are assigned with a Bayesian score. The Bayesian score corresponds to the ratio between the mature miRNA candidate's posterior probabilities for belonging to the positive versus the negative class. A ranking procedure is performed based on the assigned Bayesian score for the mature miRNAs candidates and only the top scoring candidate on each stem is assigned to the positive class.

**Figure 3:**
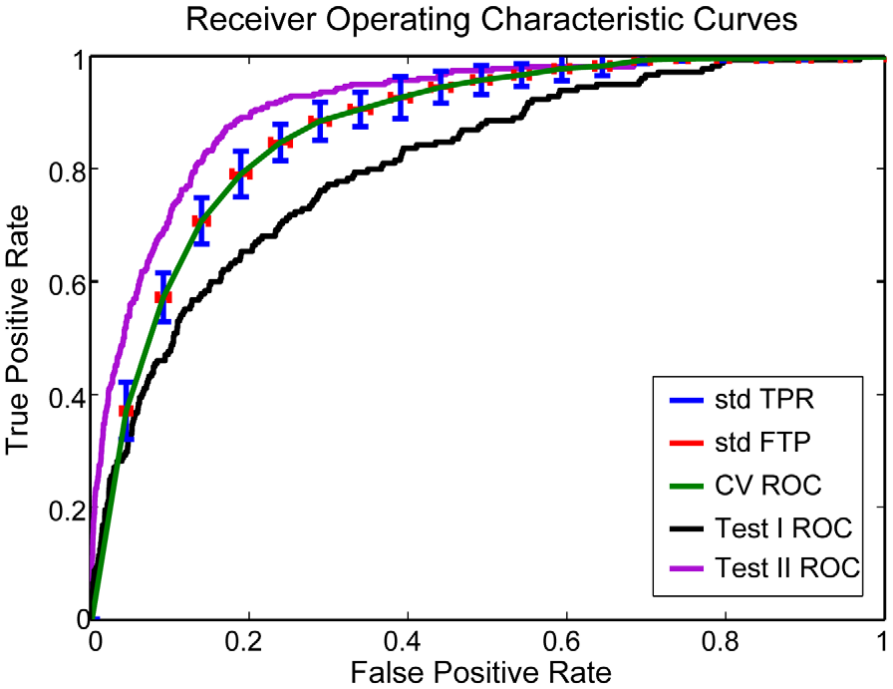
Training and generalization performance of *MatureBayes*. The average ROC curve over the 10–fold cross validation is shown in green. The standard deviation of the true positive rate (TPR) is depicted in blue while the standard deviation of the false positive rate (FPR) is shown in red. The ROC curve for the human/mouse blind test set is shown in black, while the ROC curve for the Zebrafish/*Drosophila melanogaster* blind test set is shown in purple. The average AUC for cross validation is 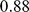, while the AUC for the human/mouse blind test set and the Zebrafish/*Drosophila melanogaster* blind test set are 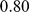 and 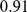, respectively.

It is important to note that classification performance is estimated based on exact match of the predicted compared to the actual mature miRNA start position. Even 1 nucleotide deviations are considered as negative examples. Figure 3 shows the Receiver Operating Characteristic (ROC) curves of the classifier for both cross validation (green) and blind test sets (purple, black). For the cross-validation curve, the standard deviation for both false and true positive rate (red and blue bars, respectively) is also provided. The Area Under the Curve (AUC) values for the cross validation (average ROC curve) and the two blind test sets are 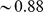, 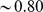 and 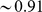, respectively. These findings show that *MatureBayes* achieves a good classification accuracy on both the training as well as the blind test set for human/mouse miRNAs and an even better performance on miRNAs from the two other species. It should be emphasized however that AUC may not be the best measure for assessing the performance of a naive Bayes classifier since the probabilities produced for negative versus positive examples can vary significantly between different precursor sequences. Thus, while positive examples may rank higher than negative examples for each precursor, the respective absolute scores which are used to generate the ROC curves do not necessarily rank higher for all positive compared to all negative examples. To address this limitation we assess our model's performance using distance distributions between the predicted and true mature miRNAs for each precursor sequence, as detailed below.

### Identification of the mature miRNA and/or the miRNA:miRNA* duplex

Although very popular, the computational discovery of novel miRNA genes is usually limited to the identification of miRNA precursor sequences [21], [36] leaving the functional part, namely the mature miRNA, unknown. To address this limitation *MatureBayes* offers the option of predicting either the strand-specific mature miRNA candidate and/or the miRNA:miRNA* duplex of each queried miRNA precursor. Prediction of strand-specific miRNA candidates is more suitable for cases where the functional strand is known *a priori*, while prediction of the miRNA:miRNA* duplex can be applied in all cases. The first is achieved by providing the top scoring mature miRNA candidate which is located on the functional strand while the latter is achieved by providing the top scoring mature miRNA candidate of the entire precursor (considering both strands) with its miRNA*. The miRNA* is defined according to [20] as the complementary, same-size mature miRNA candidate that lies on the opposite strand of the top scoring candidate with a 2 nucleotide overhang in the 3′ end. For duplex prediction, the classifier's performance is assessed assuming that the actual mature miRNA corresponds to either the predicted mature miRNA or the predicted mature miRNA*, without explicitly specifying the functional strand.

**Figure 4:**
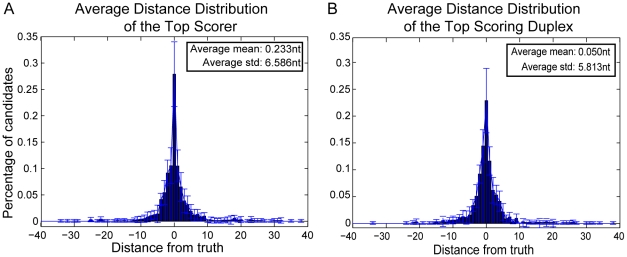
Average distance distributions of Top Scorer and Top Scoring Duplex over the 10-fold cross validation. A. The average distance distribution of the Top Scorer is estimated separately for each stem of the miRNA precursors, using only the stems that contain an actual miRNA. This approach assumes prior knowledge of the functional stem(s). B. The average distance distribution of the Top Scoring Duplex is estimated over both stems of the miRNA precursors. The distribution is generated by calculating for each precursor the distance of the actual mature miRNA(s) from the predicted candidate (miRNA or miRNA*) that is located on the same stem.

In order to assess the classifier's performance accuracy in identifying the mature miRNA and/or the miRNA:miRNA* duplex, we generate distance distributions showing the percentage of predicted candidates that are located within a specific distance from the respective actual mature miRNAs. Figure 4A shows the average distance distribution of the top scoring candidates from the actual mature miRNAs (estimated over the known functional strands) during the 10-fold cross validation procedure. The average mean of the distribution is 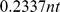, while the average standard deviation is 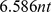. It should be noted that 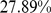 of the computational predictions match the actual miRNA start positions, while 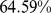 and 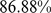 are within 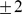 and 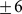 nucleotides, respectively, from the truth (see Table 1). Figure 4B shows the same distribution for the top scoring miRNA:miRNA* duplex over all precursors in the cross-validation set. The distance is measured from the start position of the actual mature miRNA, irrespectively of whether it corresponds to the predicted mature miRNA or its miRNA* candidate. If the precursor produces two mature miRNAs, both distances are calculated. The average mean of the distribution is 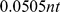 and the average standard deviation 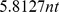. Moreover, 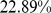 of the candidates match the actual miRNA start positions, while 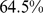 and 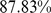 are within 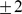 and 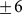 nucleotides, respectively, form the truth (see Table 1). Note that the classifier's accuracy in terms of predicting the start position of either the strand-specific mature miRNA or the miRNA:miRNA* duplex is quite similar on the cross-validation dataset.

**Table 1:** Distance distributions corresponding to Figure 4.

Distance from Truth	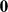	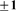	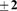	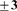	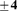	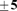	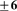	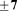	Precursors
Top Scorer ( 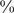 )	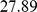	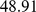	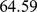	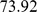	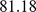	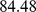	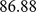	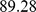	955
Top Scoring Duplex ( 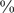 )	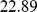	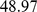	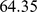	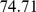	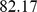	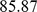	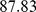	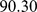	955

Table illustrating the percentages of predicted candidates that are located within 0–7 nucleotides from the start for the actual mature miRNAs for the top scoring candidate (Top Scorer) and its duplex (Top Scoring Duplex). Note that the two distributions are quite similar.

**Figure 5:**
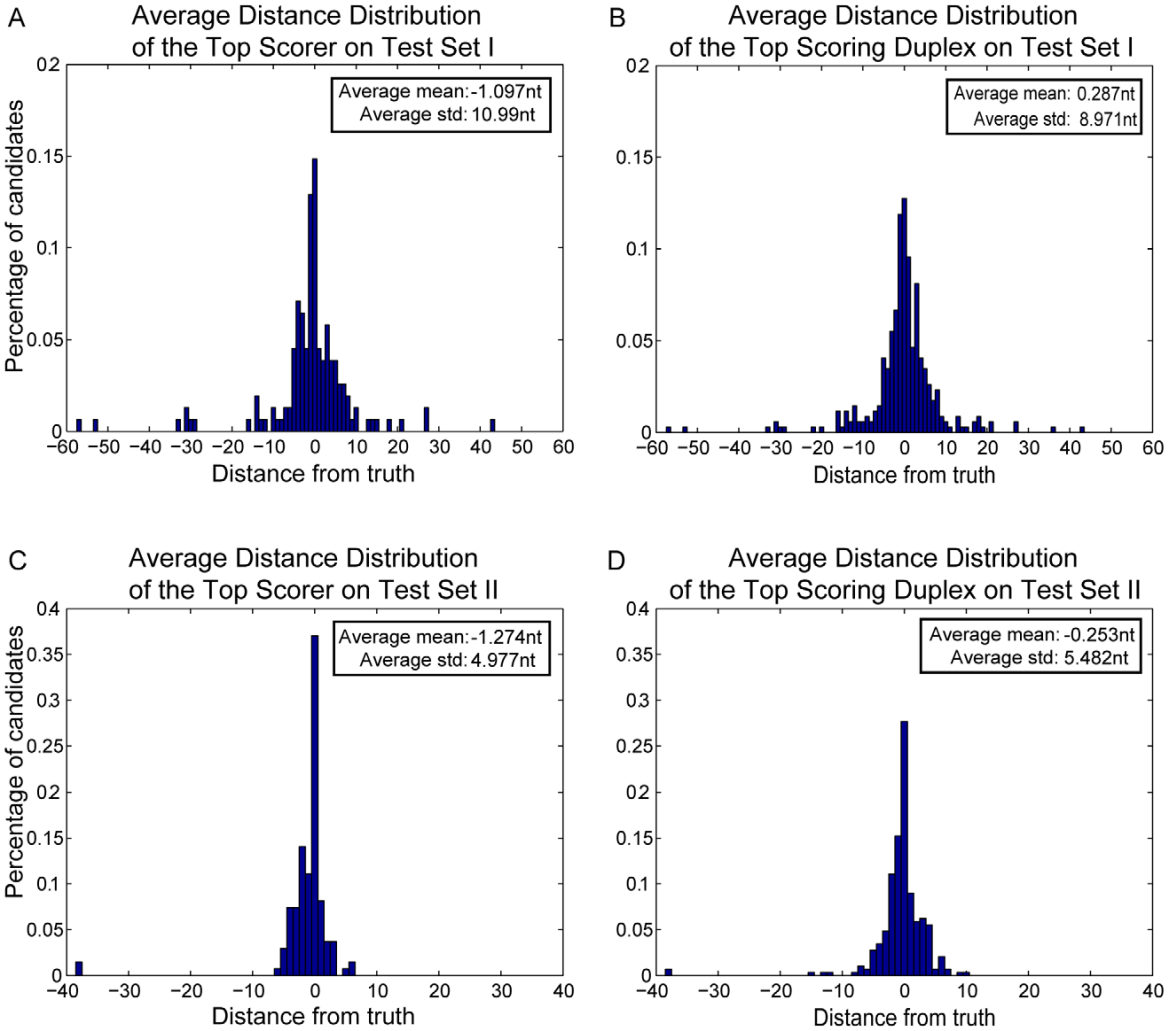
Average distance distributions of Top Scorer and Top Scoring Duplex over the two blind test sets. The distributions are generated as described in Figure 4. A,B. Human/mouse data set (Test Set I). C,D. Zebrafish/*Drosophila melanogaster* data set (Test Set II).

To assess the generalization performance of our classifier, the same distributions are also estimated for the two blind test sets as illustrated in Figure 5. Note that while the Top Scorer and Top Scoring duplex distributions are quite similar for the human/mouse test set (Test Set I), this is not the case for the Zebrafish/*Drosophila melanogaster* test set (Test Set II). In the latter, the Top Scorer has a much better prediction accuracy than the Top Scoring Duplex (see Table 2), which is also significantly larger than the performance of the classifier on the human/mouse test set. Specifically, 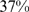 of the Top Scorer computational predictions in the Zebrafish/*Drosophila melanogaster* set match the actual miRNA start positions, while 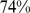 and 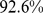 are within 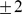 and 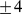 nucleotides, respectively, from the truth. The respective values for the human/mouse test set are 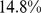, 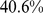 and 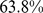. This increase in performance is probably due to the fact that the Zebrafish/*Drosophila melanogaster* set consists of mature miRNAs which have been experimentally verified in more than one species, thus forming a higher-confidence data set.

**Table 2:** Distance distributions corresponding to Figure 5.

Distance from Truth	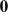	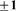	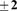	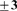	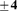	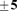	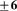	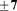	Precursors
Human and Mouse set Top Scorer ( 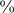 )	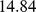	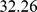	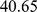	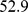	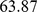	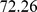	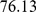	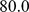	329
Human and Mouse set Top Scoring Duplex ( 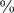 )	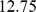	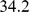	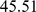	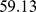	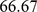	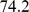	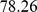	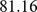	329
Zebrafish and Drosophila set Top Scorer ( 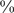 )	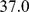	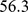	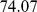	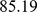	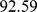	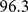	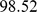	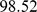	269
Zebrafish and Drosophila Top Scoring Duplex ( 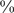 )	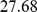	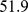	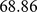	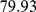	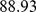	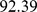	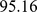	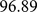	269

Table illustrating the percentages of predicted candidates that are located within 0–7 nucleotides from the start for the actual mature miRNAs for the top scoring candidate (Top Scorer) and its duplex (Top Scoring Duplex) on the two blind test sets. Note that the performance on the Zebrafish/*Drosophila melanogaster* data set is significantly larger, with more than 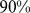 of the miRNA Top Scorer predictions located within 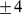 nucleotides.

### Comparison with other methods

To characterize our method's performance in comparison with existing approaches, we use a common blind test set and contrast our findings with those of two previously developed tools, namely *ProMiR* [23] and *BayesMiRNAfind* [22]. Comparison is performed separately for each tool, using the combined 329 human/mouse miRNA precursors contained in the first blind test set (Test Set I). Note that the human/mouse blind set contains precursors that were added in later versions of miRBase database (versions 11–14) and were not used to train any of the compared tools. This is not necessarily true for the second test set, thus it was not used for comparison purposes. Moreover, the similarity between the precursors in Test Set I and the ones contained in the training/validation sets is on average less than 40%. Performances are estimated only on those precursors that have been computationally predicted to contain a mature miRNA by each one of the tools, respectively. At least three more studies use computational methods to identify the mature miRNA from a miRNA precursor [24]–[26]. However, we have not been able to use those tools in our comparison analysis due to source code and data unavailability. It should also be noted that *ProMiR* and *BayesMiRNAfind* were developed with a different task in mind, specifically that of identifying the functional stem of the miRNA precursor. We compare our method against these tools to demonstrate that trivial adaptations of existing methods cannot address the problem of mature miRNA identification better than *MatureBayes*.

### Comparison with ProMiR

**Figure 6:**
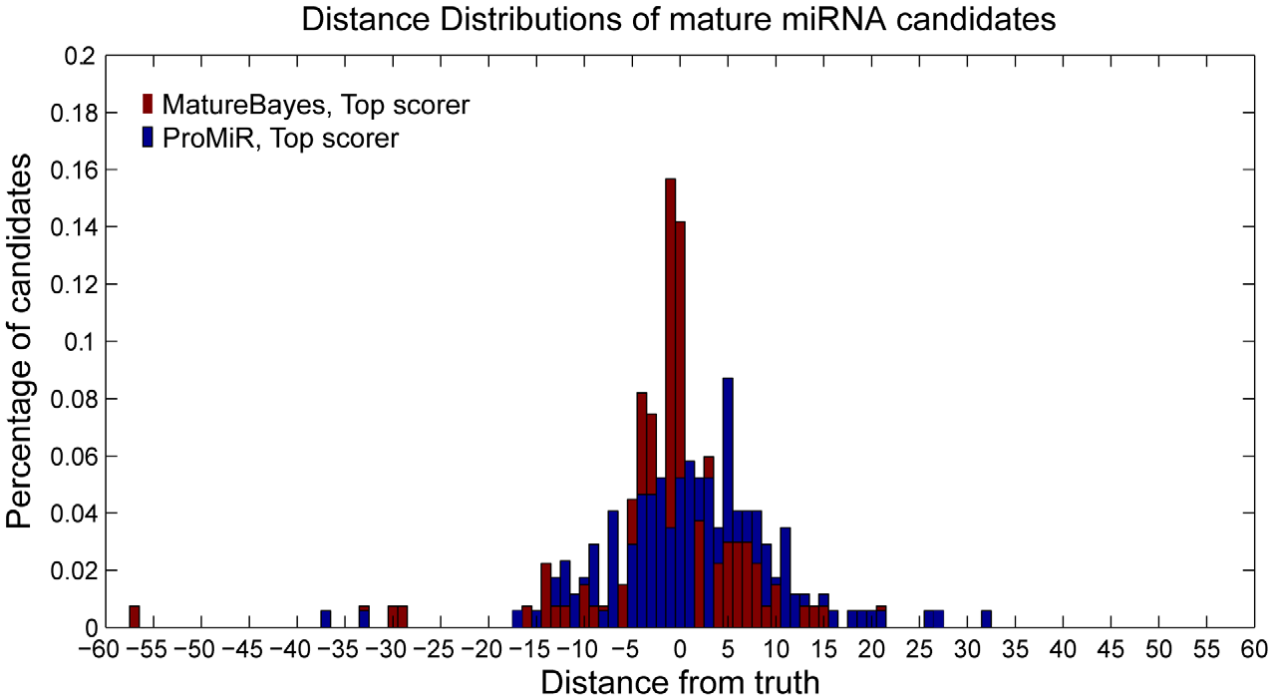
Comparison with *ProMiR*. Average distance distributions for the Top Scoring candidates provided by *MatureBayes* (red) and *ProMiR* (blue) on a common human/mouse blind test set. The set consisted of 301 miRNA precursors which were correctly predicted by *ProMiR* to contain a mature miRNA.*ProMiR* correctly identified the functional stem for 172/301, which were used for the respective distance distribution. The distance distribution for *MatureBayes* is generated using 134/301 precursors for which the correct stem was predicted, using the Top Scorer procedure. The statistical difference between the two distributions was evaluated using the Kolmogorov-Smirnov Test, confirming that the two datasets come from different distributions (p-value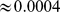).

*ProMiR* [23] implements Hidden Markov Models (HMMs) for the identification of novel miRNA precursors. Comparison with our method was performed on 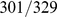 precursors which were found to contain a miRNA by *ProMiR*. Correct identification of the functional stem(s) was successful for 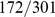 precursors by *ProMiR* versus 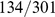 precursors by *MatureBayes*. Note that stem prediction by *MatureBayes* was achieved by selecting the stem which contained the highest scoring mature miRNA candidate. Distance distributions between the predicted and actual mature miRNA start positions were calculated for each tool, using the 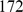 and 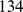 correctly predicted functional stems, respectively (see Figure 6). As shown in Figure 6 and detailed in Table 3, the start position of only 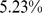 of the predicted candidates by *ProMiR* coincided with that of the respective actual miRNAs, while 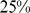 and 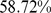 of the predictions were located within 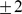 and 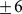 nucleotides from the truth. The respective values for *MatureBayes* were 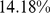, 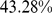 and 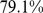, corresponding to a more than 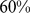 increase in performance accuracy. The statistical difference between the two distributions shown in Figure 6 was evaluated using the Kolmogorov-Smirnov Test, confirming that the two datasets belong to different distributions (p-value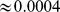). As evident from the above findings, *MatureBayes* significantly outperforms *ProMiR* in terms of predicting the start position of mature miRNA(s) within a given precursor, especially when the functional strand is known *a priori*.

**Table 3:** Distance distributions corresponding to Figure 6.

Distance from Truth	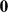	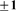	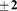	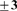	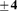	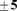	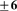	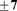	Precursors
*ProMiR* ( 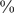 )	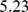	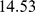	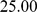	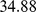	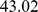	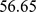	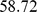	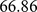	172
*MatureBayes* ( 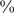 )	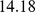	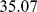	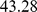	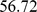	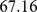	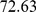	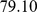	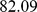	134

Table illustrating the percentage of predicted candidates which are located within a specific nucleotide distance from the actual mature miRNAs, according to the distributions shown in Figure 6.

### Comparison with BayesMiRNAfind

**Figure 7:**
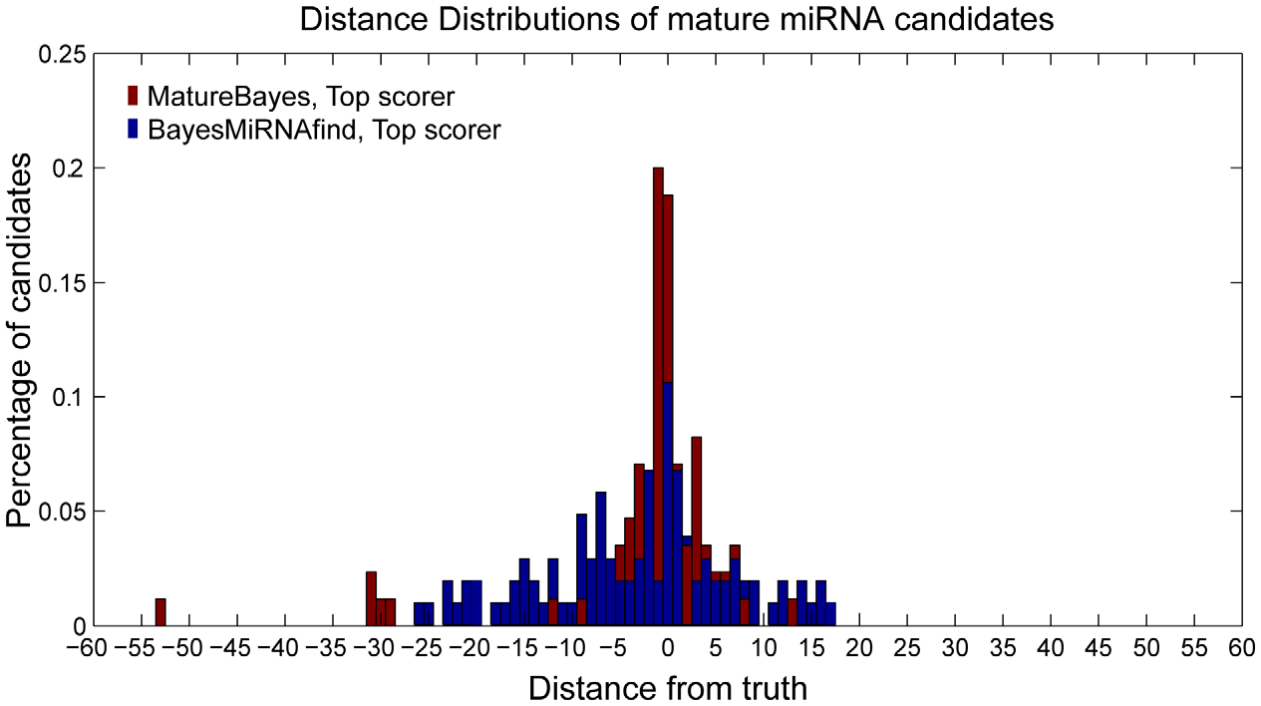
Comparison with *BayesMiRNAfind*. Average distance distributions for the Top Scoring candidates provided by *MatureBayes* (red) and *BayesMiRNAfind* (blue) on a common human/mouse blind test set. The set consisted of 181 miRNA precursors which were correctly predicted by *BayesMiRNAfind* to contain a mature miRNA. *BayesMiRNAfind* correctly identified the functional stem for 104/181, which were used for the respective distance distribution. The distance distribution for *MatureBayes* was generated using 85/181 precursors for which the correct stem was predicted, using the Top Scorer procedure. The statistical difference between the two distributions was evaluated using the Kolmogorov-Smirnov Test, confirming that the two datasets come from different distributions (p-value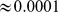).

*BayesMiRNAfind* [22] is more similar to our approach as it uses a naive Bayes classifier to predict miRNA precursors. However, it only incorporates mature miRNA prediction as a means for increasing the gene prediction performance. Comparison with our method was performed using 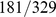 precursors in the blind test set which were found to contain a miRNA by *BayesMiRNAfind*. Correct identification of the functional stem(s) was successful for 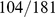 precursors by *BayesMiRNAfind* versus 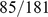 precursors by *MatureBayes*. Distance distributions between the predicted and actual mature miRNA start positions were calculated for each tool, using the 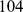 and 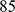 correctly predicted functional stems, respectively (see Figure 7). As shown in Figure 7 and detailed in Table 4, the start position of only 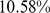 of the predicted candidates provided by *BayesMiRNAfind* coincided with that of the respective actual miRNAs, while 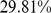 and 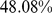 of the predictions were located within 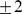 and 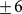 nucleotides from the truth. The corresponding values for *MatureBayes* were 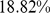, 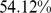 and 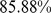, corresponding to nearly a 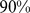 increase in performance accuracy. The statistical difference between the two distributions shown in Figure 7 was also assessed using the Kolmogorov-Smirnov Test, confirming that the two datasets come from different distributions (p-value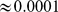).

**Table 4:** Distance distributions corresponding to Figure 7.

Distance from Truth	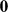	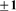	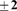	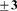	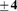	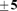	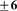	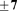	Precursors
*BayesMiRNAfind* ( 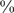 )	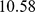	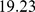	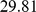	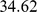	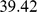	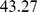	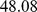	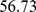	104
*MatureBayes* ( 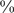 )	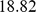	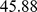	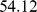	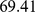	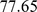	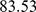	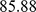	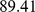	85

Table illustrating the percentage of predicted candidates which are located within a specific nucleotide distance from the actual mature miRNAs according to the distributions shown in Figure 7.

Taken together, our comparison analysis shows that (1) all three methods have a similar, poor, performance in terms of predicting the functional strand of miRNA precursors (around 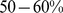) and that (2) *MatureBayes* significantly outperforms both *ProMiR* and *BayesMiRNAfind* in terms of accurately predicting the start position of a mature miRNA once the functional strand is identified. Specifically, for all deviations between 0 and 6 nucleotides, *MatureBayes* correctly identifies at least 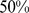 more (often double the number of) miRNAs predicted by the other tools. It should be noted that prediction of the miRNA:miRNA* duplex is an important advantage of *MatureBayes* as it avoids the problem of identifying the functional strand when this is not known *a priori* while maintaining a very similar prediction accuracy for the start position of either the mature miRNA or its miRNA*.

### Position-specific features may define Dicer recognition sites

**Figure 8:**
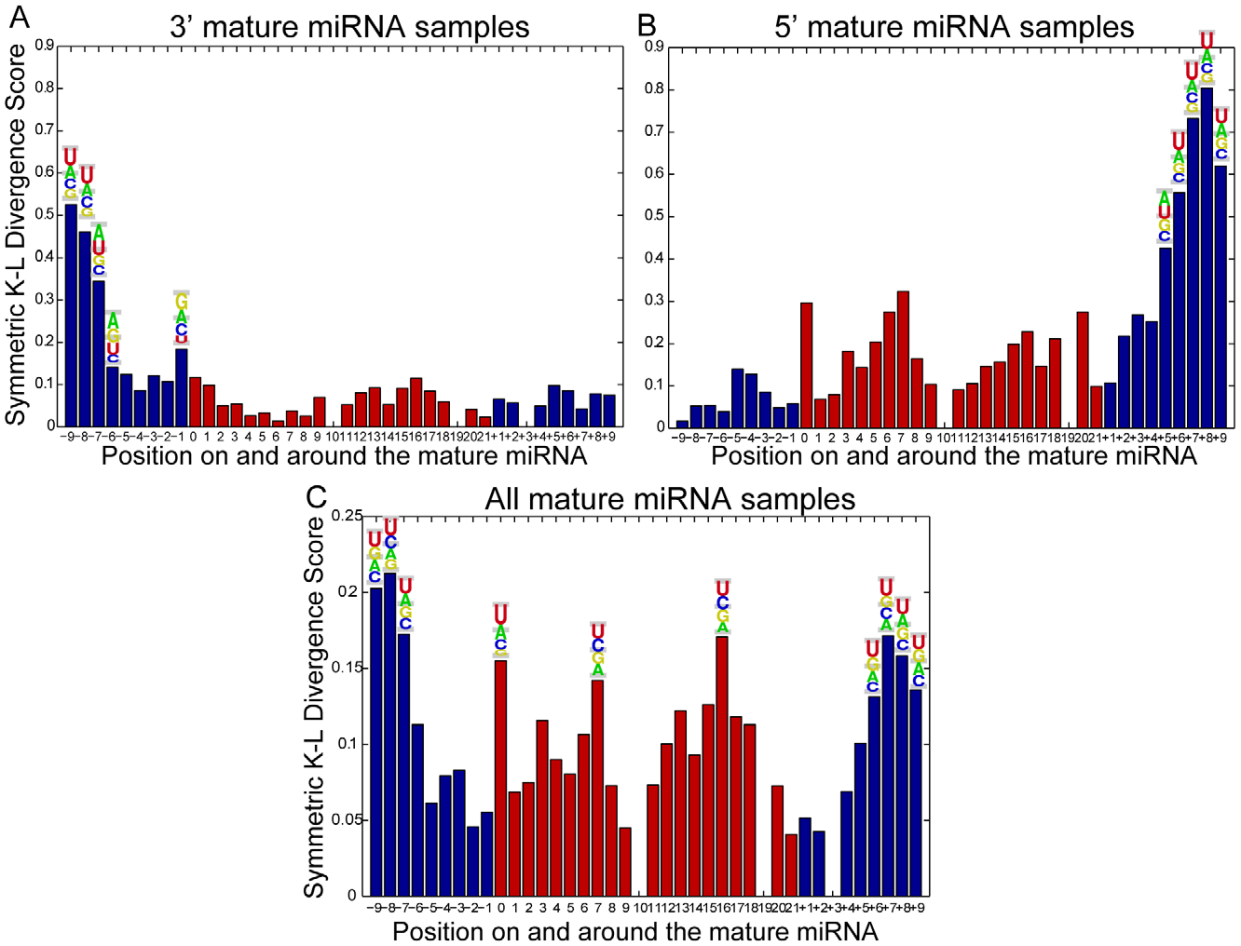
Position-specific feature distributions. All distributions are estimated according to the Kullback–Leibler divergence score over the training set. Red indicates positions within the mature miRNA while blue indicates positions surrounding the mature miRNA. A, B. Feature distributions for the 3′ and 5′ mature miRNAs respectively. C. Feature distributions for the combined data set, including both 3′ and 5′ mature miRNAs. Sequence composition information is also provided for the 10 top scoring position-specific features. Note that top scoring features tend to cluster in positions 7–9 nucleotides *before* the start position of the mature miRNA for 3′ samples and *after* the 22nd nucleotide (representing the average end position) of the mature miRNA for 5′ samples. For the combined data set shown in C, the most informative position-specific features lie symmetrically in both ends of the mature miRNA flanking regions. All of the above features are most likely to contain a U base except the 7th position in 3′ samples where the probability of containing Adenine is slightly higher.

As with all classification methods, high performance is most likely to result from the high discriminatory power of specific input features representing key sequence and structural characteristics of miRNA precursors. Moreover, such features may represent a recognition signal for mature miRNA cleavage by the Dicer complex. To investigate this hypothesis we further analyze the 38 features utilized by the optimal *MatureBayes* classifier. These include: (a) 37 position-specific features containing combined sequence and structure information of each position and (b) the distance between the start position of the mature sample and the closest end of the nearest precursor hairpin as it folds into a secondary structure. The K-L score distributions for all selected position-specific features along with the sequence probabilities for the top 10 of these features are shown in Figure 8. Figures 8A and 8B show the distributions over the 3′ and 5′ mature miRNA samples respectively, while Figure 8C shows the combined distribution estimated over all mature miRNAs in the training set. As evident from the individual distributions, the most informative features tend to cluster in positions 7–9 nucleotides *before* the start position of the mature miRNA for 3′ samples and *after* the 22nd nucleotide (corresponding to the average end position) of the mature miRNA for 5′ samples. Since we use the combined set of both 3′ and 5′ samples for feature selection, the most informative position-specific features as shown in Figure 8C lie symmetrically in both ends of the flanking regions surrounding the mature miRNA. Importantly, all of the 10 top scoring features in the combined dataset are very likely to contain a U base. Moreover, the 7–9 nucleotide triplets in both 3′ and 5′ samples are also very likely to consist of Uracil (except the 7th position in 3′ samples where the probability of containing Adenine is slightly higher). Statistical comparison between the sequence composition distributions of true miRNAs and negatives for these positions was inconclusive (only position 8 after the end of the mature miRNA had a p value larger than 0.001), suggesting that a larger dataset is needed to verify the possible existence of a ‘UUU’ signal.

**Figure 9:**
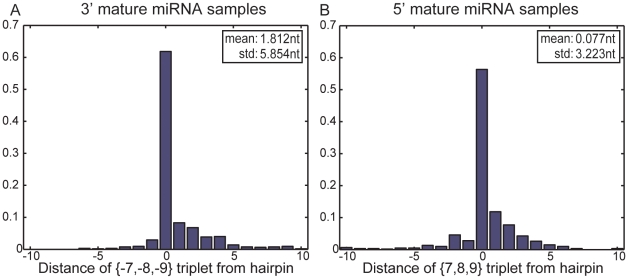
Distance distributions for position 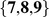 triplets for the 3′ and 5′ mature miRNAs, respectively. For 3′ mature miRNAs, the triplet located at positions 7,8 and 9 nucleotides *after* the 22nd position is used. All distributions are estimated over the training set. A distance equal to 0 denotes that the triplet is part of the loop while a distance of 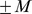 nucleotides denotes that the triplet starts/ends at M nucleotides from the start (or the end, for the opposite strand) of the loop. Note that in both cases, position 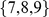 triplets are located inside the hairpin (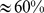 of the triplets) or in very close proximity to the start of the hairpin (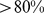 of the triplets is within 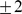 nucleotides from the hairpin).

To further investigate the potential role of position 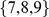 triplets in determining the mature miRNA start position, we generate distance distributions of the respective triplets in both 3′ and 5′ mature samples. For 3′ mature miRNAs, we use the triplet located *prior* to the start position, while for 5′ samples we use the triplet located *after* the 22nd position. Figure 9 shows the distance distributions of each triplet from the two ends of the closest hairpin loop. Distance 0 denotes that the triplet is part of the loop while a distance of 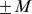 nucleotides denotes that the triplet starts/ends at M nucleotides from the start (or the end, for the opposite strand) of the loop. As evident from the figure, position 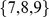 triplets are located within or very close to the adjacent hairpin loop. Specifically, approximately 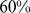 of the triplets are located inside the hairpin for both 3′ (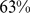) and 5′ (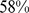) mature miRNA samples, while 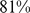 and 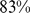 of the triplets are located within 2 nucleotides from the hairpin and 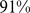 and 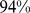 of the triplets are located within 5 nucleotides from the hairpin for 3′ and 5′ samples, respectively. Moreover, statistical analysis of the (a) combined structure and sequence distributions as well as (b) the structure distributions alone between the positive and negative classes showed that position 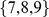 triplets are significantly different (Smirnov-Kolmogorov test, 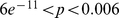) between the two classes, further supporting their discriminatory role.

**Figure 10:**
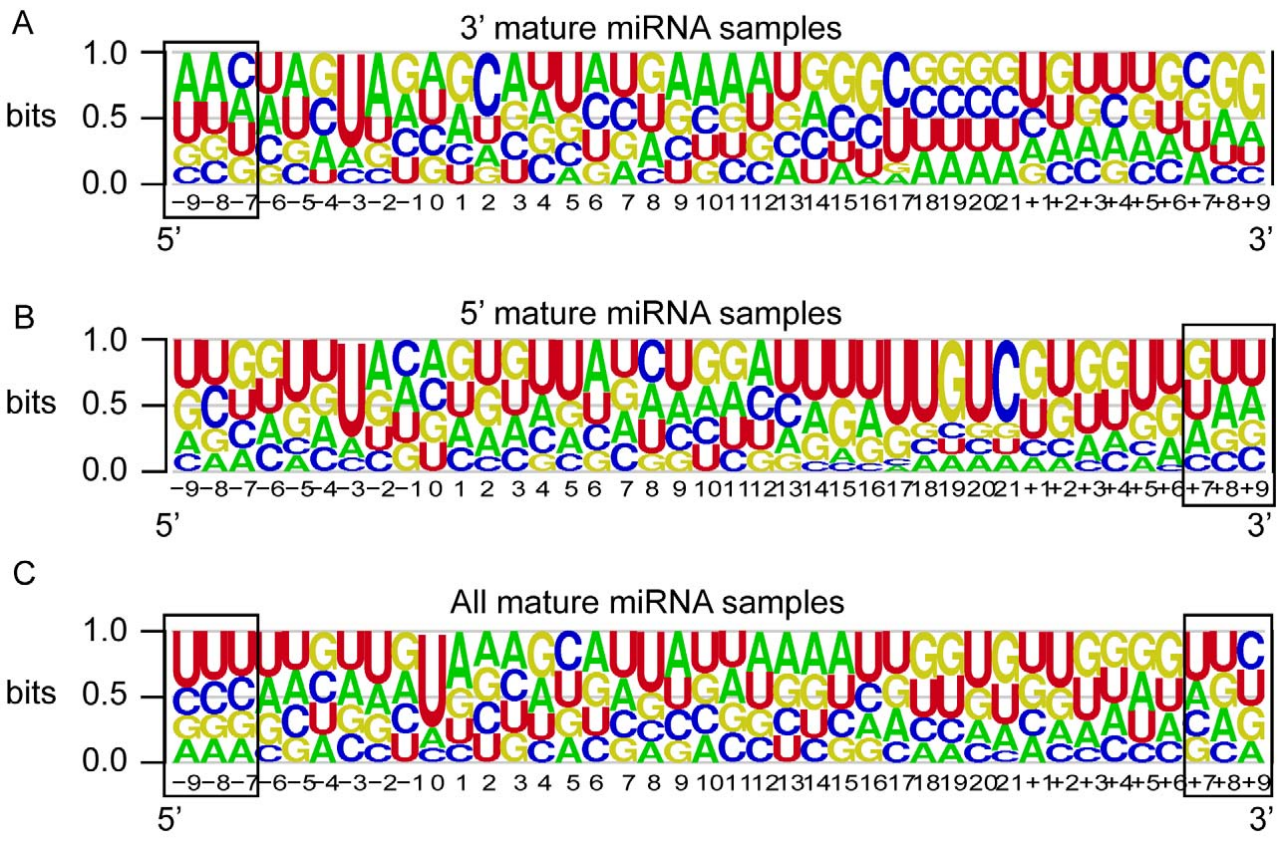
Sequence composition information of the Zebrafish/*Drosophila melanogaster* test set. The sequence composition is along the mature miRNAs and their flanking regions. A, B. Sequence composition for the 3′ and 5′ mature miRNAs, respectively. C. Sequence composition for the combined data set, including both 3′ and 5′ mature miRNAs. Note that position 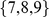 triplets (*prior* to the mature miRNA for 3′ samples, *after* the mature miRNA for 5′ samples and symmetrically at both ends of the mature miRNA for all samples) in this dataset are also very likely to contain Uracil, as was the case with the human/mouse training set shown in Figure 8.

Taken together, our findings show that positions 7,8, and 9 from the start (for 3′ samples or the end for 5′ samples) of the mature miRNA appear to be relatively conserved in terms of their base composition (likely to contain Uracil) as well as their structural characteristics (all three are most likely to be inside the hairpin loop). These findings suggest that the first few bases within or in close proximity the hairpin may serve as a recognition signal for Dicer and associated proteins, thus determining the start position for both 3′ and 5′ mature miRNAs. Interestingly, this feature appears to also be present in miRNAs from the two other species tested. As shown in Figure 10, a similar pattern of sequence composition is observed in positions 7–9 nucleotides *before* the start position of the mature miRNA for 3′ samples, *after* the 22nd nucleotide of the mature miRNA for 5′ samples and symmetrically in both ends of the mature miRNAs for the combined set. In all cases their is a relatively high probability that position 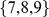 triplets in miRNAs from Zebrafish and *Drosophila melanogaster* contain a Uracil. While this suggest the possible existence of a general rule for Dicer processing that applies for multiple organisms and not just mammalian precursors, a larger dataset is needed to verify that sequence composition at positions 7–9 nucleotides serves as the primary recognition signal for Dicer. On the other hand, the statistically significant difference between the positive and negative structure distributions for the same positions, along with their presence inside or in close proximity to the hairpin loop, indicates that the recognition signal for Dicer processing may be the lack of base pairing upstream of the mature sequence, and not so much the sequence composition.

## Discussion

In this work we address the problem of identifying the starting nucleotide of mature miRNA(s) that are produced by mammalian (human and mouse) miRNA precursors. Using a simple statistical classifier, namely the Naive Bayes Classifier (NBC), and taking into account sequence as well as structural information of miRNA precursors, our tool can predict the start position of the mature miRNA and/or the miRNA:miRNA* duplex with high accuracy, significantly outperforming two existing methods. Important advantages of our method in addition to high performance include the requirement of relatively small amounts of training data to estimate the classifiers parameters as well as a direct intuition about the importance of the features used. Our tool is provided both as a user friendly trainable interface as well as a web-based scanning application (http://mirna.imbb.forth.gr/MatureBayes.html) which can either be used independently or as part of a pipeline when querying novel miRNA precursors provided by the sister software SSCprofiler [37] found at http://mirna.imbb.forth.gr/SSCprofiler.html. In all cases, the user has a large degree of flexibility in terms of dataset specification and parameter tuning.

Our method works by combining information about the sequence and structure of each nucleotide position along the entire length of mammalian miRNA precursors. We show that the integration of such biological features with previously identified characteristics of mature miRNAs such as the distance from the closest hairpin [24], [27], can significantly enhance prediction accuracy. Interestingly, we find that the most informative position-specific features are located in the flanking region surrounding the mature miRNA. Specifically, the highest scoring features are consistently found in positions 7–9 nucleotides from the start (for 3′ samples) or the end (for 5′ samples) of the mature miRNA, which are either inside or in very close proximity to the closest hairpin loop. Moreover, these triplets have a relatively high probability of containing a U base, in both 3′ and 5′ samples and their secondary structure characteristics are significantly different between the positive and negative classes, suggesting that they may serve as a recognition signal for accurate cleavage by Dicer. Importantly this position-specific ‘UUU’ feature is also present in the miRNAs from Zebrafish and *Drosophila melanogaster*, indicating the possible existence of a more general rule for Dicer processing. Finally, the distance between the start position of the mature miRNA and the closest hairpin is also very important for accurate miRNA identification, suggesting that true mature miRNAs are located in specific positions independently of their length or the actual size of the precursor.

An important advantage of our method compared to existing tools is the use of negative data which are generated from the same precursors that contain the true mature miRNAs. This process was selected as it closely resembles the challenges faced by experimentalists when discovering a new miRNA gene. Most of the computational tools that can be used to predict the functional part of the miRNA precursor estimate their performance accuracy in terms of true positive rate alone, ignoring entirely the false positive rate [25], [26]. It is a matter of semantics as well as a great challenge to define a true negative example when it comes to mature miRNAs. However, a major issue in such a classification task is not only to maximize the tool's ability to identify true positives but also to minimize the false positive rate. In an effort to combine both of these criteria, we use experimentally verified human and mouse miRNA precursors to generate positive and negative examples and then train and evaluate the performance of our classifier measured as both the Area Under the ROC Curve (AUC) and the distance of the predicted miRNA start position from the truth.

The effectiveness of *MatureBayes* in recognizing mature miRNAs in both human and mouse precursors was demonstrated using a blind set of 329 recently identified precursors added in versions 11–14 of miRBase. The method reached a prediction accuracy of 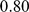 measured as the Area Under the ROC Curve (AUC). More importantly, we show that our tool's performance, measured as the distance of the predicted from the actual mature miRNA, significantly outperforms two existing tools. The percentage of mature miRNA candidates provided by *MatureBayes* that are located within 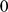, 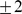 and 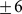 nucleotides from the truth is approximately double compared to that of *BayesMiRNAfind* and over 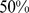 larger than *ProMiR* predictions for the same distance. Overall, in comparison to both methods, a significantly larger portion of our predicted candidates is located within a few nucleotides from the actual mature miRNA(s). Moreover, our tool can avoid the problem of identifying the functional strand in novel miRNA precursors, where the performance accuracy of all compared tools is very poor, by providing as computational truth the miRNA:miRNA* duplex while maintaining the same high accuracy in terms of start nucleotide prediction.

The ability of our method to identify the start position of mature miRNAs from other organisms was assessed using a high-confidence blind test set of 269 precursors from Zebrafish and *Drosophila melanogaster* in which all mature miRNAs have been experimentally verified in more than one organism. The method reached a prediction accuracy of 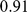 measured as the Area Under the ROC Curve (AUC), which is significantly larger than the respective performance on human/mouse miRNAs. Moreover, the tool's performance, measured as the distance of the predicted (Top Scorer) from the actual mature miRNA, was also significantly larger on this data set. These findings show that although trained on human/mouse miRNAs, our method has a very good generalization performance on data from at least two other species (Zebrafish and *Drosophila melanogaster*).

In conclusion, our findings suggest that position specific sequence and structure information and the distance of the starting position from the hairpin combined with a simple Bayes classifier achieve a very good performance on the challenging task of mature miRNA identification. More importantly, we suggest the possible existence of a recognition signal for accurate cleavage which is located within the hairpin loop, in close proximity for the mature miRNA sample.

## Supporting Information

Table S1 The AUC of the average ROC curve, over the 10-fold cross validation, of the best naive bayes classifiers for every combination of flanking region and scanning window.
(0.03 MB PDF)

Table S2 The AUC of the average ROC curve, over the 10-fold cross validation procedure, for naive bayes classifiers trained with flanking region 9nt.
(0.04 MB PDF)
