# Supplementary material for: MatureBayes: A Probabilistic Algorithm for Identifying the Mature miRNA within Novel Precursors
Source: PLoS One. 2010 Aug 6;5(8):e11843. doi: 10.1371/journal.pone.0011843 (PMC2917354; doi:10.1371/journal.pone.0011843)
Supplement: Table S1 — The AUC of the average ROC curve, over the 10-fold cross validation, of the best naive bayes classifiers for every combination of flanking region and scanning window. (0.03 MB PDF) [file pone.0011843.s001.pdf]

Supplementary Table S1.

Table 1: The AUC of the average ROC curve, over the 10–fold cross validation, of the best naive bayes classifiers for every combination of flanking region and scanning window.

**Flanking Region**	**Window 18nt**	**Window 20nt**	**Window 22nt**	**Window 24nt**
**0nt**	0.8629	0.8615	0.8621	0.8624
**3nt**	0.8671	0.8658	0.8675	0.8661
**5nt**	0.8597	0.8614	0.8662	0.8642
**7nt**	0.8592	0.8630	0.8716	0.8696
**9nt**	0.8599	0.8673	**0.8771**	0.8704
**12nt**	0.8585	0.8691	0.8745	0.8658
