# Supplementary material for: MatureBayes: A Probabilistic Algorithm for Identifying the Mature miRNA within Novel Precursors
Source: PLoS One. 2010 Aug 6;5(8):e11843. doi: 10.1371/journal.pone.0011843 (PMC2917354; doi:10.1371/journal.pone.0011843)
Supplement: Table S2 — The AUC of the average ROC curve, over the 10-fold cross validation procedure, for naive bayes classifiers trained with flanking region 9nt. (0.04 MB PDF) [file pone.0011843.s002.pdf]

Supplementary Table S2

Table 1: The AUC of the average ROC curve, over the 10–fold cross validation procedure, for naive bayes classifiers trained with flanking region 9nt.

**Number of *Position Oriented* Features**	**Window 18**	**Window 20**	**Window 22**	**Window 24**
1	0.8445	0.8423	0.8418	0.8420
2	0.8455	0.8534	0.8430	0.8431
3	0.8434	0.8408	0.8405	0.8392
4	0.8439	0.8432	0.8465	0.8443
5	0.8471	0.8468	0.8500	0.8482
6	0.8454	0.8469	0.8518	0.8456
7	0.8551	0.8570	0.8512	0.8557
8	0.8577	0.8598	0.8631	0.8584
9	0.8557	0.8586	0.8618	0.8517
10	0.8511	0.8561	0.8601	0.8524
11	0.8520	0.8569	0.8608	0.8529
12	0.8529	0.8588	0.8627	0.8543
13	0.8540	0.8591	0.8636	0.8548
14	0.8567	0.8616	0.8661	0.8577
15	0.8578	0.8640	0.8686	0.8601
**16**	**0.8599**	0.8634	0.8676	0.8593
17	0.8540	0.8644	0.8693	0.8612
18	0.8548	0.8631	0.8392	0.8607
19	0.8540	0.8629	0.8691	0.8614
20	0.8544	0.8632	0.8694	0.8610
21	0.8558	0.8636	0.8696	0.8614
22	0.8555	0.8640	0.8703	0.8632
23	0.8562	0.8637	0.8711	0.8631
24	0.8568	0.8648	0.8715	0.8637
25	0.8577	0.8654	0.8721	0.8654
26	0.8583	0.8662	0.8727	0.8661
27	0.8549	0.8666	0.8728	0.8663
28	0.8567	0.8651	0.8744	0.8669
29	0.8555	0.8667	0.8732	0.8672

Continued on next page

Table 1 – continued from previous page

**Number of *Position Oriented* Features**	**Window 18**	**Window 20**	**Window 22**	**Window 24**
30	0.8565	0.8658	0.8747	0.8691
31	0.8561	0.8668	0.8741	0.8685
32	0.8571	0.8670	0.8758	0.8695
33	0.8572	0.8671	0.8759	0.8686
**34**	0.8562	**0.8673**	0.8759	0.8702
**35**	0.8558	0.8662	0.8760	**0.8704**
36	0.8550	0.8655	0.8762	0.8703
**37**	-	0.8651	**0.8771**	0.8690
38	-	0.8641	0.8762	0.8698
39	-	-	0.8757	0.8690
40	-	-	0.8753	0.8685
41	-	-	-	0.8690
42	-	-	-	0.8686
